# Mild Phenotype in a Patient with a *De Novo* 6.3 Mb Distal Deletion at 10q26.2q26.3

**DOI:** 10.1155/2015/242891

**Published:** 2015-07-29

**Authors:** George A. Tanteles, Elpiniki Nikolaou, Yiolanda Christou, Angelos Alexandrou, Paola Evangelidou, Violetta Christophidou-Anastasiadou, Carolina Sismani, Savvas S. Papacostas

**Affiliations:** ^1^Clinical Genetics Department, The Cyprus Institute of Neurology and Genetics and Archbishop Makarios III Medical Centre, 2370 Nicosia, Cyprus; ^2^Clinical Sciences Neurology Clinic B, The Cyprus Institute of Neurology and Genetics, 2370 Nicosia, Cyprus; ^3^Cytogenetics and Genomics Department, The Cyprus Institute of Neurology and Genetics, 2370 Nicosia, Cyprus

## Abstract

We report on a 29-year-old Greek-Cypriot female with a *de novo* 6.3 Mb distal 10q26.2q26.3 deletion. She had a very mild neurocognitive phenotype with near normal development and intellect. In addition, she had certain distinctive features and postural orthostatic tachycardia. We review the relevant literature and postulate that certain of her features can be diagnostically relevant. This report illustrates the powerful diagnostic ability of array-CGH in the elucidation of relatively mild phenotypes.

## 1. Introduction

Distal deletions of the long arm of chromosome 10 are uncommon. Since the first published report of an individual with a distal 10q deletion in 1978 [1], there have been over 30 cases with deletions involving either the 10q26.2 or the 10q26.3 breakpoint reported in the literature. The exact locations and sizes of distal 10q deletions vary and involve either the terminal or subterminal region of chromosome 10q [2].

Deletions or duplications of the telomeric region of chromosome 10q have not previously been associated with a clearly recognizable phenotype. However, patients with a 10q monosomy present with certain clinical characteristics which include facial dysmorphism, congenital heart defects, and varying degrees of developmental delay and intellectual disability [2–5]. Other common findings include strabismus, neurobehavioral manifestations, and urogenital anomalies [2, 5, 6].

We report on a 29-year-old lady with a 6.3 Mb* de novo* distal 10q26.2q26.3 deletion associated with a mild phenotype which includes distinctive facial features, strabismus, tachyarrhythmia, and relatively mild cognitive issues.

## 2. Patient and Methods

### 2.1. Clinical Description

Review of the family history revealed that the proband was the third child born to nonconsanguineous parents, a 32-year-old father and a 27-year-old mother. Her father had a history of type II diabetes while her mother underwent ablation for ventricular ectopics. Her younger brother also had a history of tachyarrhythmias and her eldest sister was affected with Turner syndrome. She was born following a normal vaginal delivery at 37-week gestation with a birth weight of 2.6 kg. Soon after birth, she developed jaundice and severe hyperbilirubinemia for which she required exchange transfusion thrice. In addition, she had unspecified breathing difficulties requiring NICU admission for approximately three weeks. She had congenital strabismus for which she underwent corrective surgeries at the ages of 2.5 years and 15 years. There were no other major issues reported during childhood. Developmentally, there were no concerns and she completed a senior high school without requiring support. She described herself as an average pupil and there was no clear evidence of significant learning issues. Her motor and intellectual developments were reported as normal.

She presented at the age of 21 years with recurrent episodes of brief sudden loss of consciousness. During such episodes, she would lose muscle tone and fall without injuring herself. In their maximum frequency, these episodes occurred 2-3 times daily but subsequently became less frequent occurring at least once or twice per week. The patient did not report any associated bladder or bowel control loss or postepisode confusion. Most of the times, these episodes followed stressful events or could occur when she became tired. On a few occasions, blurring of vision could precede the episode. In addition to these paroxysmal episodes, she had a history of iron deficiency anaemia.

**Figure 1:**
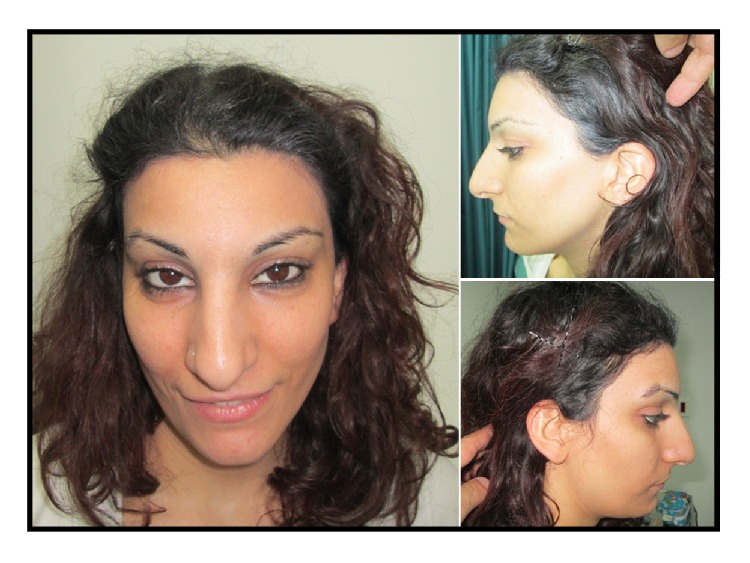
Frontal and profile views of our patient.

**Figure 2:**
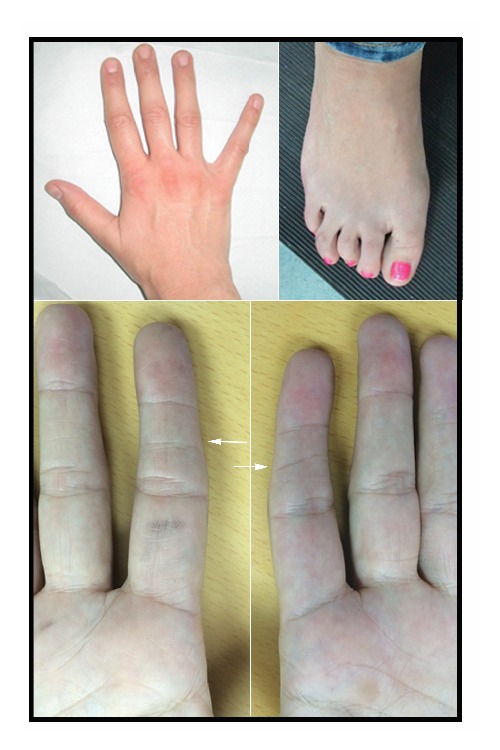
Hand and foot views. Note the curled toes and in particular the distinctive bilateral transverse creases involving the middle phalanx of the index finger (white arrows).

On examination, at the age of 29 years, her height was 155 cm (9th centile), her weight was 57 kg (25th–50th centile), and her occipitofrontal circumference (OFC) was 54 cm (25th–50th centile). She had distinctive facial features with upslanting palpebral fissures, a long tubular nose with a prominent nasal bridge and tip, an overhanging columella, and a short philtrum. She had right-sided strabismus and asymmetry of the angles of the mouth with retrognathia. She had a left-sided posteriorly rotated ear with an overfolded helix. She had a small hypomelanotic macule over the left shoulder and 2-3 small hypomelanotic macules over the right shoulder. She had transverse creases in the mid phalanges of the index fingers bilaterally. She had curled 2nd, 3rd, and 4th toes on the right (Figures 1 and 2). Her patellae were present. Cardiovascular and neurological examinations were otherwise unremarkable.

During follow-ups, the patient reported occasional episodes of palpitations for which she remained under the care of a cardiologist, while her episodes of loss of consciousness subsequently resolved. The patient seemed to have very few problems with regard to her independence and could manage her finances and personal hygiene without any difficulties. She obtained a college degree as a nursery teacher, could hold a job, and drive a car.

Previous normal or negative investigations included a full blood count, biochemistry, liver and thyroid function tests, plasma lactate, CK, a basic metabolic screen, vitamin B12, folate, molecular test for fragile X syndrome, an abdominal ultrasound scan, and an ophthalmology evaluation including visual evoked potentials. A brain MRI scan revealed small white matter lesions at the trigons of the lateral ventricles. Several EEGs did not reveal any evidence of epileptiform activity. Echocardiography, cardiac electrophysiological studies, and abdominal ultrasound scans were unremarkable. Tilt table test was positive for postural orthostatic tachycardic syndrome with normal cardiac anatomy.

On the basis of her clinical presentation and facial features, array-CGH analysis was requested. Informed consent was obtained by the patient.

### 2.2. Cytogenetic and Molecular Analyses

DNA of the index patient and her parents was isolated from peripheral blood using the QIAamp DNA Midi Kit (Qiagen, Hidden, Germany) according to the supplier's protocol. Array-CGH (comparative-genomic-hybridization) was carried out using the Cytochip ISCA array (BlueGnome, version 1.0) with 180,000 oligos in a 4 × 180 k format according to the recommendations of the manufacturer. Briefly, 500 ng of patient and pooled reference gDNA were differentially labeled using the Bioprime DNA Labeling System (Invitrogen, Carlsbad, CA) and the Cy3 and Cy5 fluorescent dyes (GE Healthcare, UK, Ltd.), respectively. Hybridization was carried out using an automated slide processor HS 400 PRO Hybridization Station (Tecan Inc., Männedorf, Switzerland). The array was scanned at 3 um resolution using the Agilent DNA microarray scanner (Agilent Technologies Inc., Santa Clara, CA, USA) and fluorescent ratios were calculated using the BlueFuse Multi software V4.2 (BlueGnome Ltd., Cambridge, UK). Fluorescence* in situ* hybridization (FISH) analysis was carried out on metaphase preparations using subtelomeric specific probes for the short (p-arm) and long arms (q-arm) of chromosome 10 (Cytocell, Cambridge, UK) according to the recommendations of the manufacturer.

## 3. Results

**Figure 3:**
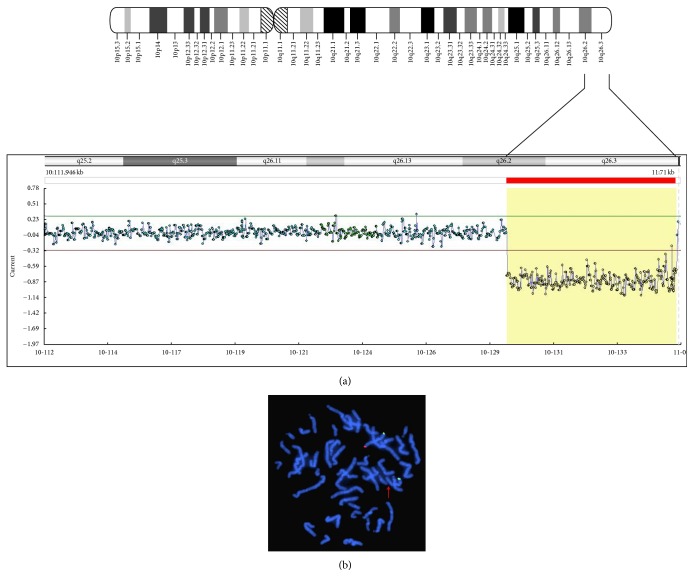
(a) Array-CGH analysis result demonstrating the 6.3 Mb distal deletion of chromosome 10 (q-arm) at chromosomal band 10q26.2 extending to band 10q26.3. (b) FISH analysis of the index patient's metaphases showing two signals of the 10p region and only one signal of the 10q region.

Array-CGH analysis revealed a distal deletion of approximately 6.3 Mb in size on the long arm of chromosome 10 (q-arm) at chromosomal band 10q26.2 extending to band 10q26.3 (location 129,142,062-135,434,149 using build GRCh37 (hg19)). The region spans approximately 6.3 Mb and encompasses several known genes (DECIPHER: https://decipher.sanger.ac.uk/) including* CALY* and partially deleted* DOCK1*. In order to confirm the deletion, FISH analysis showed positive 10ptel (green) and 10qtel signals (red) on the normal chromosome 10 but only a positive 10ptel (green) signal on the del(10) (Figure 3), therefore confirming the distal deletion on the long arm of chromosome 10 detected by array-CGH analysis. Furthermore, FISH analyses were performed for both parents to determine whether the deletion originated from any chromosomal rearrangement is present in one of the parents. Parental FISH analyses showed normal hybridization pattern on both chromosomes 10, indicating that the deletion originated in the patient as a* de novo *event.

## 4. Discussion

Constitutional subterminal 10q deletions are rare and attempts to correlate deletion size to phenotype have been reported. These deletions have been associated with a wide range of clinical findings including facial dysmorphism, learning difficulties, varying degrees of developmental delay, and intellectual disability as well as cardiovascular abnormalities [5, 7]. Other common findings include strabismus, urogenital anomalies, cryptorchidism in males, and behavioral problems [3, 5–7]. In this report, we describe a female patient with a* de novo* distal 10q26.2q26.3 deletion of approximately 6.3 Mb who presented with distinctive facial features, strabismus, postural orthostatic tachycardic syndrome, and relatively mild cognitive deficits. Despite the fact that no formal neurocognitive evaluation was performed (including IQ measurement), her social and practical skills, including communication, social interaction, taking care of her personal hygiene, managing her finances, effective use of independent transportation, level of independence, fine and gross motor skills, are evidence of normal or near normal development and intellect.

The wide variability in phenotypic expression of 10q deletions has been suggested to correlate to deletion size [3, 4]. However, there does not seem to be clear evidence to support this observation [2]. The variable degree of intellectual disability also seems to exhibit great interfamilial variability and this has been shown in familial cases that share the same deletion [3, 5].

Interrogation of the Database of Genomic Variants revealed that the deleted region encompasses 35 genes, 23 of which are listed in OMIM. There were very few candidates for specific correlations with the patient's phenotype. Eight genes namely,* DOCK1*,* PPP2R2D*,* BNIP3*,* DPYSL4*,* INPP5A*,* GPR123*,* ADAM8*, and* CALY*, are notable as they have been associated with certain clinical manifestations (Table 1). The* CALY *gene encodes a single transmembrane protein expressed in the brain which is involved in vesicle trafficking-related functions, important for efficient synaptic transmission in the central nervous system [2]. Deletion of* CALY* has been reported in patients with behavioral problems and it has been suggested that haploinsufficiency of this gene could partially contribute to the development of behavioral disturbances [2]. In addition, deletions of the* INPP5A *and* GPR123* genes, which encode proteins involved in central nervous system development, could lead to neurobehavioral abnormalities [2]. Furthermore, it has been suggested that* DPYSL4* gene deletion could be associated with behavioral problems and/or intellectual disability because its function is involved in neuronal differentiation [2, 3, 5]. The* ADAM8* gene encodes a protein that is implicated in neurogenesis and muscle development and has been associated with susceptibility to autism [8]. According to Iourov et al., haploinsufficiency of* PPP2R2D* and* BNIP3* is likely to cause a phenotypic effect such as developmental delay or intellectual disability [4]. Our patient had near normal intelligence and did not exhibit significant neurobehavioral issues. Although our patient's phenotype could not be supported by the published literature as described above, her presentation could reflect variability in phenotypic expression as it has been suggested that alterations in genes involved in signaling and regulation pathways could be responsible for the wide degree of phenotypic variability observed in patients with 10q deletions [7]. Finally, the phenotypic heterogeneity could result from incomplete penetrance or due to other genetic, epistatic, epigenetic, or environmental factors.

The* DOCK1* gene (partially deleted in our patient) is known to be involved in several biological processes including cell migration and phagocytosis [7].* DOCK1 *haploinsufficiency can result in craniofacial dysmorphism and cardiac and urinary anomalies [8]. There is a hypothesis which suggests that a critical region containing the* DOCK1* gene could cause the characteristic clinical phenotype seen in 10q deletions [2]. It remains plausible that haploinsufficiency of* DOCK1* could be associated with our patient's distinctive facial features. In addition to the facial features, of note are the distinctive bilateral transverse creases involving the middle phalanx of the index finger which we postulate that it could represent a diagnostic clue. She also had a history of postural orthostatic tachycardic syndrome, the significance of which remains unclear. The positive family history of arrhythmias could however point towards an independent to the 10q26.2q26.3 deletion, factor as potentially causal.

In conclusion, a female with a* de novo* 6.3 Mb distal deletion of chromosome 10q at chromosomal band 10q26.2 extending to 10q26.3 displaying a very mild phenotype compared to previous reports of similar deletions is reported. She had near normal development and intellect, postural orthostatic tachycardic syndrome and certain distinctive features. This report illustrates the powerful diagnostic ability of array-CGH in the elucidation of relatively mild phenotypes. Further molecular studies should be performed to further establish the genotype-phenotype correlations of these deletions with a view to clarify the role and influence of the genes involved in this region.

## Figures and Tables

**Table 1:** OMIM genes located in the deleted 10q26.2q26.3 region.

Gene symbol	Gene title	Function	OMIM
*DOCK1 *	Dedicator of cytokinesis 1	Cell migration, phagocytosis [7]	601403
*PPP2R2D *	Protein phosphatase 2, regulatory subunit B, and delta	Cause of developmental delay or intellectual disability [4]	613992
*BNIP3 *	BCL2/adenovirus E1B 19 kDa interacting protein 3	Cause of developmental delay or intellectual disability [4]	603293
*DPYSL4 *	Dihydropyrimidinase-like 4	Neuronal differentiation [3, 5]	608407
*INPP5A *	Inositol polysphosphate-5-phosphatase	Central nervous system development [2]	600106
*GPR123 *	G protein-coupled receptor 123	Central nervous system development [2]	612302
*ADAM8 *	ADAM metallopeptidase domain 8	Neurogenesis, muscle development [7]	602267
*CALY *	Calcyon neuron-specific vesicular protein	Vesicle trafficking-related functions [2]	604647

OMIM: Online Mendelian Inheritance in Man.
